# Reducing exposure to COVID-19 by improving access to fever clinics: an empirical research of the Shenzhen area of China

**DOI:** 10.1186/s12913-021-06831-4

**Published:** 2021-09-13

**Authors:** Qing Yong, Dinglong Liu, Guoqi Li, Wanshan Wu, Wenjie Sun, Sijing Liu

**Affiliations:** 1 School of Resources and Environmental Engineering, Wuhan University of Technology, No. 122, Luoshi Road, Hongshan District, Wuhan, Hubei Province People’s Republic of China; 2 Wuhan Transportation Planning and Design Co., Ltd., Huijin Plaza, 1268 Jinghan Avenue, Jiang’an District, Wuhan, Hubei Province People’s Republic of China; 3 School of Transportation and Logistics, Southwest Jiaotong University, Chengdu, People’s Republic of China

**Keywords:** COVID-19, Fever clinics, SARS-CoV-2, Accessibility, Travel time

## Abstract

**Background:**

The current 2019 coronavirus disease (COVID-19) pandemic is hitting citizen’s life and health like never before, with its significant loss to human life and a huge economic toll. In this case, the fever clinics (FCs) were still preserved as one of the most effective control measures in China, but this work is based on experience and lacks scientific and effective guidance. Here, we use travel time to link facilities and populations at risk of COVID-19 and identify the dynamic allocation of patients’ medical needs, and then propose the optimized allocation scheme of FCs.

**Methods:**

We selected Shenzhen, China, to collect geospatial resources of epidemic communities (ECs) and FCs to determine the ECs’ cumulative opportunities of visiting FCs, as well as evaluate the rationality of medical resources in current ECs. Also, we use the Location Set Covering Problem (LSCP) model to optimize the allocation of FCs and evaluate efficiency.

**Results:**

Firstly, we divide the current ECs into 3 groups based on travel time and cumulative opportunities of visiting FCs within 30 min: Low-need communities (22.06%), medium-need communities (59.8%), and high-need communities (18.14%) with 0,1–2 and no less than 3 opportunities of visiting FCs. Besides, our work proposes two allocation schemes of fever clinics through the LSCP model. Among which, selecting secondary and above hospitals as an alternative in Scheme 1, will increase the coverage rate of hospitals in medium-need and high-need communities from 59.8% to 80.88%. In Scheme 2, selecting primary and above hospitals as an alternative will increase the coverage rate of hospitals in medium-need and high-need communities to 85.29%, with the average travel time reducing from 22.42 min to 17.94 min.

**Conclusions:**

The optimized allocation scheme can achieve two objectives: a. equal access to medical services for different types of communities has improved while reducing the overutilization of high-quality medical resources. b. the travel time for medical treatment in the community has reduced, thus improving medical accessibility. On this basis, during the early screening in prevention and control of the outbreak, the specific suggestions for implementation in developing and less developed countries are made.

**Supplementary Information:**

The online version contains supplementary material available at 10.1186/s12913-021-06831-4.

## Background

Inequitable allocation of medical resources is the most serious problem under the outbreak and will lead to higher mortality rates [1]. The epidemic has continued to spread in Brazil, India, Bangladesh, and Africa [2], making the allocation of medical resources such as FCs more urgent and severe.

In response to the transmission of COVID-19, China has adopted various public health interventions to control the spread of the epidemic, including case isolation and contact tracing measures, intensive intracity and intercity traffic restriction, social distancing measures, centralized quarantine, and improvement of medical resources [2, 3]. One of the effective measures is the establishment of a therapeutic mechanism and diversion mechanism for patients with fever or respiratory symptoms [4], which aims to separate potentially infectious from non-infectious patients [5] with the purpose of not only reducing the risk of cross-infection but also improving the utilization of medical resources [4]. However, after the outbreak, the severity of SARS-CoV-2 pneumonia poses a great strain on critical care resources in hospitals, especially if they are not adequately staffed or resourced [6, 7]. And the number of patients (both suspected and confirmed) greatly exceeded the number of physicians [4]. Now, most countries in the world face problems such as the inadequate and unbalanced supply of medical services [8, 9]. Inadequate access to readily available healthcare has been shown to influence many health activities and outcomes, including higher mortality [1, 10–12], lower rates of follow-up [13], increased infectious disease morbidity [14, 15]. However, about 60% of suspected cases remain asymptomatic coronavirus carriers in urban communities with uneven distribution of medical resources, and risk of transmission in which is not low [16–19], posing a great threat to the lives of urban residents, doctors and patients [20, 21].

Developing equitable health systems is at the core of the United Nations Sustainable Development Goals [22, 23]. However, in most countries of the world, inequalities in socio-economic status and housing conditions are often also reflected in health inequalities [10, 24, 25]. To prevent, detect, and respond to the pandemic, we require access to timely and high-quality healthcare from strong and resilient health systems [26–28]. Among them: improving the accessibility of health facilities, quickly connecting patients with the community health system [15], and ensuring equal access to treatment for all is the key to winning this battle.

**Fig. 1:**
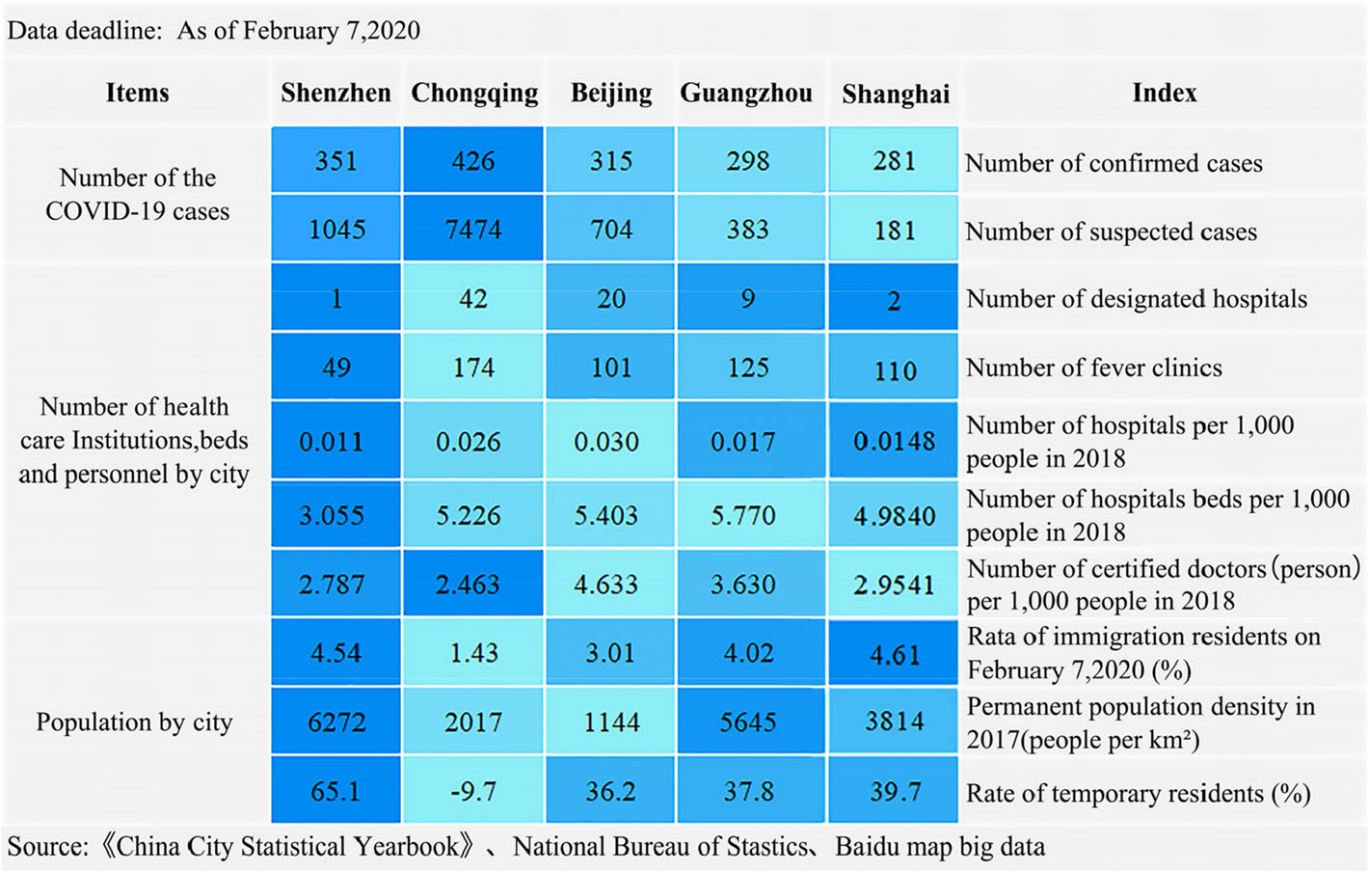
Infections numbers and medical facilities in the five cities during the COVID-19 outbreak

As of February 7, 2020, a total of confirmed cases of a novel coronavirus (COVID-19) in china were 34,546, 722 deaths. In the early stages of the outbreak, considering the data availability, we selected four traditional Chinese first-tier cities (Beijing, Shanghai, Shenzhen, and Guangzhou) and the only municipality in the western region (Chongqing) as the potential research subjects, as shown in Fig. 1. The ultimate subjects of the study were identified through a preliminary analysis of the three aspects of the urban epidemic situation, population and medical facilities.

As of February 7, Shenzhen has 351 confirmed cases and 1045 suspected cases in total, after Chongqing’s 426 confirmed cases. In addition, Shenzhen is a city with the highest population density (6727 people per km 2), highest rate of temporary residents (65.1%), and highest rate of immigration residents (4.54%), where the population is dense with active movement and complex composition. In contrast, Shenzhen has only one designated hospital (The third people’s hospital of Shenzhen), which can provide up to 500 beds in specific wards, as well as 49 FCs so far; At the same time, the number of hospitals, hospital beds in 2018, and practicing (assistant) doctors in 2018 all came last in all cities, accounting respectively for 36.7, 52.9, and 60.2% of the highest cities in China. To prevent another outbreak, based on data availability, this paper investigates the dynamics of the medical accessibility of Shenzhen residents 1 week after the peak period.

**Fig. 2:**
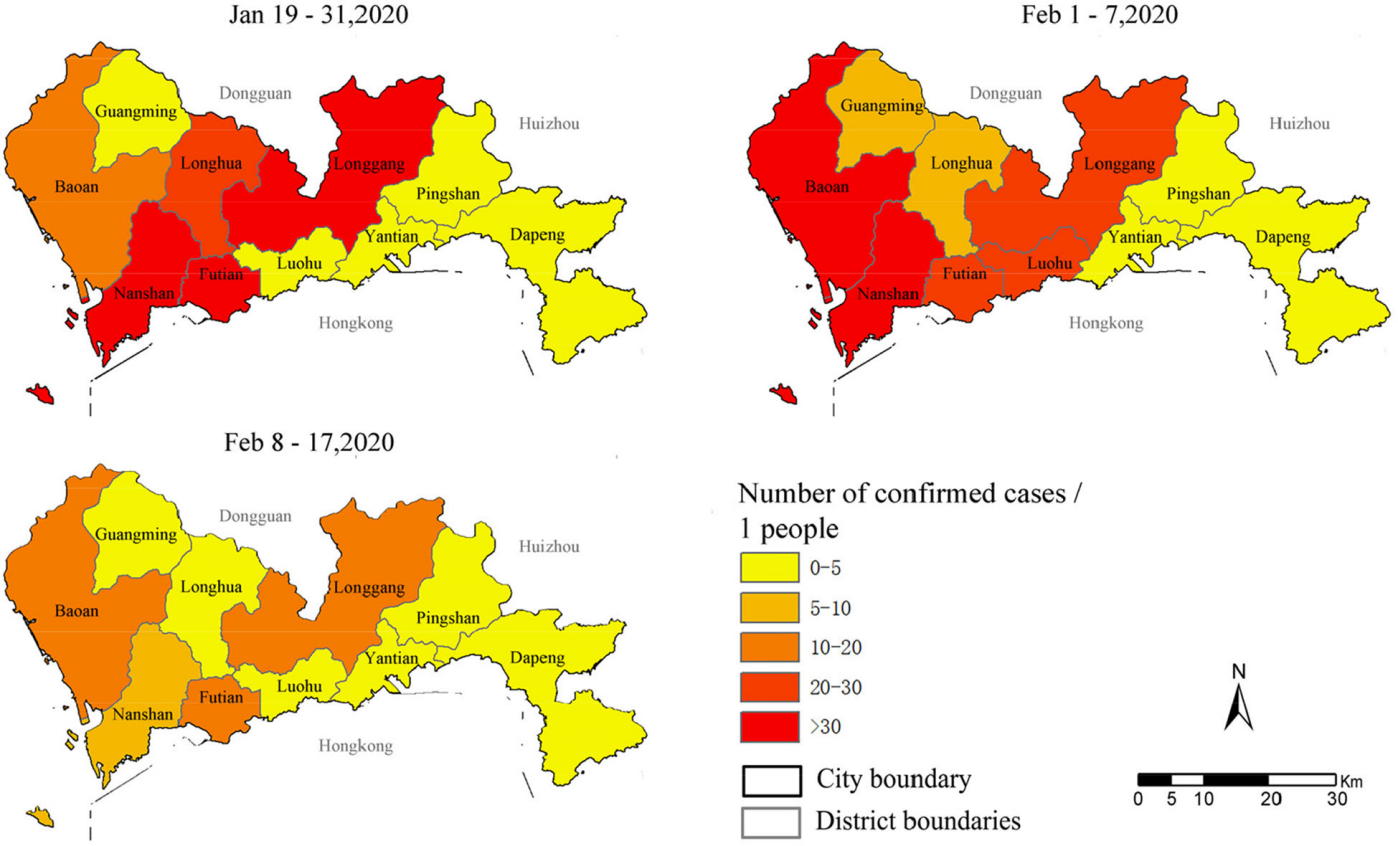
The Geographic Distribution of Daily Rates of COVID-19 Cases Across the 3 Periods in Shenzhen, China. The daily rate of cases is expressed as the number of confirmed cases per day per 1 person, grouped by each of the 10 districts of the city of Shenzhen

The study was conducted in Shenzhen (Fig. 2), Guangdong Province, where the first confirmed case occurred on January 19, and the number of confirmed cases increased rapidly daily, reaching a peak on January 31. During that time, the ECs were mainly concentrated in the Nanshan District, Futian District, and Longgang District of Shenzhen, with a relatively high incidence of epidemics in the central urban areas. In the latter week, the epidemic gradually spread to the suburbs, with marked geographical differences in the confirmed cases. Finally, with the implementation of travel restrictions and border control measures, the condition for COVID-19 improved slightly and there were no new confirmed cases in the city as of 18 February.

In this study, in order to truly reflect the variability in the medical accessibility under different travel modes for residents in Shenzhen, we used Baidu map real-time navigation data instead of road network calculation theory [29–31] to obtain the travel time and define health facility access. What’s more, travel time can be used to define accessibility in this paper due to it not only directly determines the risks of virus infection but also a key indicator in related predictions studies, such as H7N9 Avian Influenza [31], African Malaria [32], African Food security [33]. Next, accessibility measurements have been widely applied to many fields including resident commuting, public resource allocation, and urban management among the elderly and low-income groups [34–37], but few studies have addressed the optimal allocation of fever clinics. For these reasons, we first analyzed the two travel modes for visiting FCs in Shenzhen, driving, and non-driving mode, and compared the differences in the availability of it. Then, we further measure variability in visiting FCs by using travel time to link facilities and populations at risk of COVID-19 and illustrate the rationality of the location of FCs. Finally, we built the Location Set Covering Problem (LSCP) based on the accessibility and cumulative opportunities of visiting FCs to balance the rational allocation of medical resources, and proposed two alternative dynamic adjustment schemes for FCs.

## Methods

For our analyses, we used two data sources and two analysis methods: (1) Data of COVID-19 situation and medical resource; (2) Baidu navigation data; (3) The calculation of cumulative opportunities of visiting FCs; (4) Location Set Covering Problem model.

### Data 1: the number of FCs and beds in designated hospitals, and the situation of the COVID-19 epidemic

Based on the data of COVID-19 patients and medical resources publicly released by the Shenzhen Health Commission (http://wjw.sz.gov.cn/) as of February 29, 2020, including the daily-added confirmed cases (Fig. S1), the released ECs, 49 FCs, and 1 designated hospital, we identify the location of ECs and FCs using Baidu maps. This paper selects epidemic data 1 week after the peak of the epidemic including the number of newly confirmed cases and newly added ECs (Fig.S2), and the spatial distribution maps of ECs, FCs (Fig.S3). Furthermore, the calculation of available beds is based on the standard that designated hospitals can provide up to 500 beds in special wards. The attribute data of FCs mainly includes name, address, type, level of the hospital, and so on.

The ECs are mainly distributed in Nanshan (48), Futian (41), Longgang (37), and Baoan District (29), accounting for 76%. In terms of the level of epidemic hospitals, there are 10 secondary hospitals and 35 tertiary hospitals, accounting for 20 and 71.4% respectively, are mainly concerned at Longgang District, Baoan District, Futian District, and Nanshan District.

### Data 2: Baidu navigation data

To truly represent the travel time to health facilities, based on the origin/destination(O/D) point of 204 ECs and N FCs, we acquired the travel time of visiting FCs in three modes of walking, public transportation and driving using real-time navigation data obtained from the Baidu Map API (http://lbsyun.Baidu.com/). In accordance with the principle of visiting the nearest FCs, we compute the shortest time to visit FCs under driving mode and non-driving mode combining walking and public transportation (Fig.S4). In addition, we evaluated the transportation accessibility of the two modes.

### Methods S1:the calculation of cumulative opportunities for visiting FCs

This paper generates the OD time matrix *T*_*ij*_(204 × *n*) based on the travel time *t*_*ij*_ between regions. The threshold range for travel time is below 15, 15–30, 30–45, and above 45. Observing whether the travel time *t*_*ij*_ satisfies the threshold value to generate the cumulative opportunities of visiting FCs, the formula is as follows:

S2a $$ {C}_{ijk}=\sum \limits_{j\in V}{X}_{ijk},\forall i\in U,k\in \left\{1,2\right\} $$

S2b $$ {X}_{ijk}\in \left\{0,1\right\},\kern0.5em \forall i\in U,j\in V,\kern0.5em k\in \left\{1,2\right\} $$

S2c $$ {t}_{ij}\in {T}_{ij},\kern0.5em \forall i\in U,\kern0.5em j\in V $$

Where.

*U*= A set of 204 urban ECs, community *i* ∈ *U*;

*V*= A set of FCs in Shenzhen, fever clinic *j* ∈ *V*;

*k*= Two modes of visiting FCs for community residents, which takes a value of 1, if visiting in non-driving mode, otherwise it is 2; 

*X*_*ijk*_= A 0-1 variable, which assumes a value of 1 if the travel time from a community *i* to hospital *j* in *k* mode meets the threshold range, otherwise, it is 0.

*C*_*ijk*_= Cumulative opportunities of visiting FCs in mode *k* within a community *i*, reflecting the perfect degree of the medical facilities around the community.

## Methods S2:location set covering problem model

To improve the utilization efficiency of high-quality medical resources and shorten the gap in visiting opportunities and transportation accessibility between different ECs, this paper employs the Location Set Covering Problem (LSCP) model to optimize the allocation of FCs [38, 39] which is as follows:

S3a $$ M\mathrm{in}\ \sum \limits_{\mathrm{j}\in \mathrm{v}}{A}_j $$

S3b $$ \mathrm{s}.t.\kern0.5em \sum \limits_{i\in U}{X}_{ij}\le {A}_j\ast W,\kern0.5em \forall j\in V $$

S3c $$ \sum \limits_{j\in V}{X}_{ij}\ge 1,\forall i\in U $$

S3d $$ {\displaystyle \begin{array}{l}{A}_j\in \left\{0,\kern0.5em 1\right\},\kern0.5em \forall j\in V\\ {}{X}_{ij}\in \left\{0,\kern0.5em 1\right\},\kern0.5em \forall i\in U,j\in V\end{array}} $$

Where.

*W* = Infinite positive number.

*A*_*j*_=A 0-1 variable, which assumes a value of 1 if a hospital *j* is determined to be a fever clinic for a community *i.* otherwise, it is 0.

*X*_*ij*_= A 0–1 variable, which assumes a value of 1 if the shortest travel time from a community *i* to a hospital *j* exceeds 30 min, otherwise, it is 0.

The objective function (Eq. (3a)) attempts to cover the maximum number of ECs with the minimum number of facilities. Eq. (3b) assumes that if the shortest time-consuming from the community *i* to the hospital *j* does not exceed 30 min, the hospital *j* is selected as a fever clinic for treatment in the community *i*. Eq. (3c) means that each epidemic community must be covered by just one fever clinic.

## Results

### Measures of transportation accessibility for ECs under different transport modes

Referring to the accessibility-based measurements proposed in *Hulland and Van Wee* [40], this paper reckoned cumulative opportunities for visiting FCs based on the travel time of ECs [41]. Stemming from 2016 urban resident trip surveys in Shenzhen, more residents prefer traveling in non-driving models, accounting for 74%. But it is safer and faster to drive during the outbreak of the epidemic. Therefore, taking 15 min, 30 min, and 45 min as a measurement index, we calculated the cumulative opportunities of visiting FCs under the two models.

**Fig. 3:**
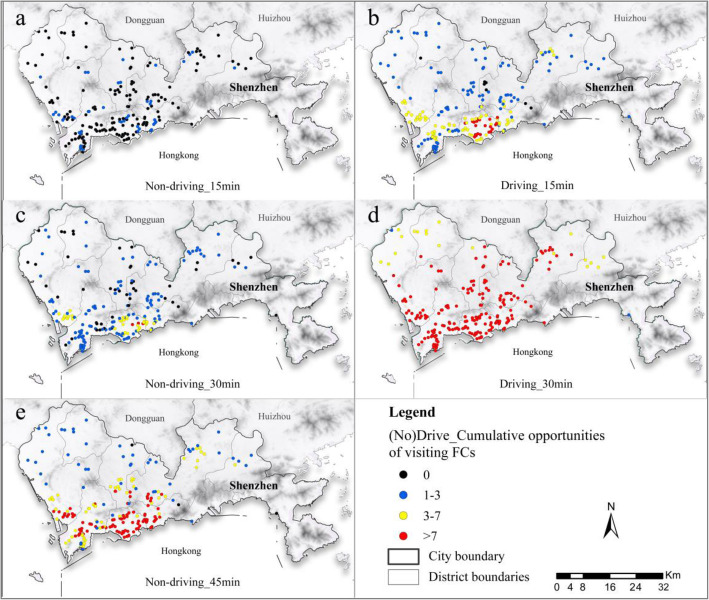
Number of FCs accessible by ECs under different travel modes and time constraints. **a, b** respectively represents the number of accessible FCs under the non-driving and driving modes with a travel time limit of 15 min (inclusive). **c, d** respectively demonstrates the number of accessible FCs under non-driving and driving modes with a travel time limit of 30 min (inclusive). **e** manifests the number of accessible FCs under non-driving modes with a travel time limit of 45 min (inclusive)

Figure 3a, b shows that within 15 min, 77.45% of the ECs traveling in non-driving mode could not arrive on time to the nearest FCs, while only 3.43% in driving mode could not reach. Most of these communities are located near district boundaries, such as the west of Longgang District, the east of the Yantian District, and the south of Longhua District. Figure 3b, e shows traveling in driving mode within 15 min is equivalent to traveling in non-driving mode within 45 min. Similarly, the number of inaccessible ECs and FCs is very close between the two models, accounting for 3.43 and 1.47%, respectively; Still, there are 45 communities accounting for 22.06% that cannot reach the nearest hospital in the non-driving mode while people there can achieve it by driving within 30-min travel thresholds (Fig. 3c, d).

On the whole, traveling in non-driving mode means more travel time, resulting in some people turning to seek treatment in non-government designated medical institutions. This way of treatment is not conducive to timely diagnosis and follow-up for patients, thereby increasing the risk of prevention and control.

### Rationality evaluation of medical resources

According to the plan of the National Health Commission of the PRC, by 2020, China will achieve a 30-min primary medical service circle. Bosanac [42] advocated a similar criterion, so this paper investigated the cumulative opportunities of visiting FCs under the non-driving mode in ECs within 30 min, to explore the balance between medical resources needs and supplies in each district.

**Fig. 4:**
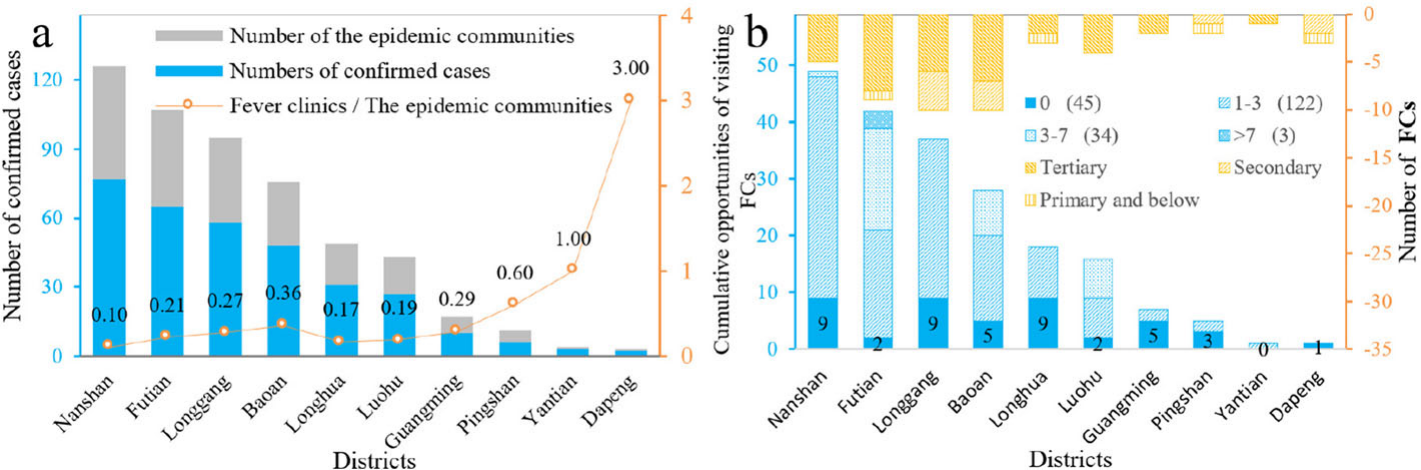
**a** Numbers of confirmed cases, numbers of the ECs and numbers of FCs in Shenzhen (Data source: Shenzhen Municipal Health Commission.); **b** Cumulative opportunities of visiting FCs based on 30-min travel thresholds and number of FCs in Shenzhen (summarized by hospital levels) (Data source: Baidu map navigation data)

To begin with, we explored the current status of medical resources in all the districts. Figure 4a shows that the number of FCs in the first 4 districts with the severe epidemic in Shenzhen, is the opposite. Especially there are only 5 FCs in Nanshan District with the lowest average number of clinics owned by the ECs, only 0.1, followed by Longhua and Luohu District of 0.17 and 0.19. Then the significant contradiction and imbalance between supply and demand of medical resource allocation are further revealed based on the cumulative opportunities of visiting FCs. Figure 4b indicates that 45 communities with 0 opportunities of visiting FCs are mainly distributed in Nanshan, Longgang, Longhua, Baoan, and Guangming Districts. In mainland China, FCs affiliated with the emergency departments (EDS), the setting it is related to hospital service capacity (standard). Of FCs released by the Shenzhen government, the tertiary hospitals secondary, primary, and below hospitals are accounted for 71.4, 20.4, and 8.2% respectively. Moreover, about 62% of FCs in secondary and above hospitals are located in Futian, Longgang, and Baoan Districts. Thanks to that the initial site selection scheme was constrained by the service capacity and location of graded hospitals, it was difficult to achieve equitable access to medical treatment for residents in different communities, thus the location of FCs was not reasonable.

**Fig. 5:**
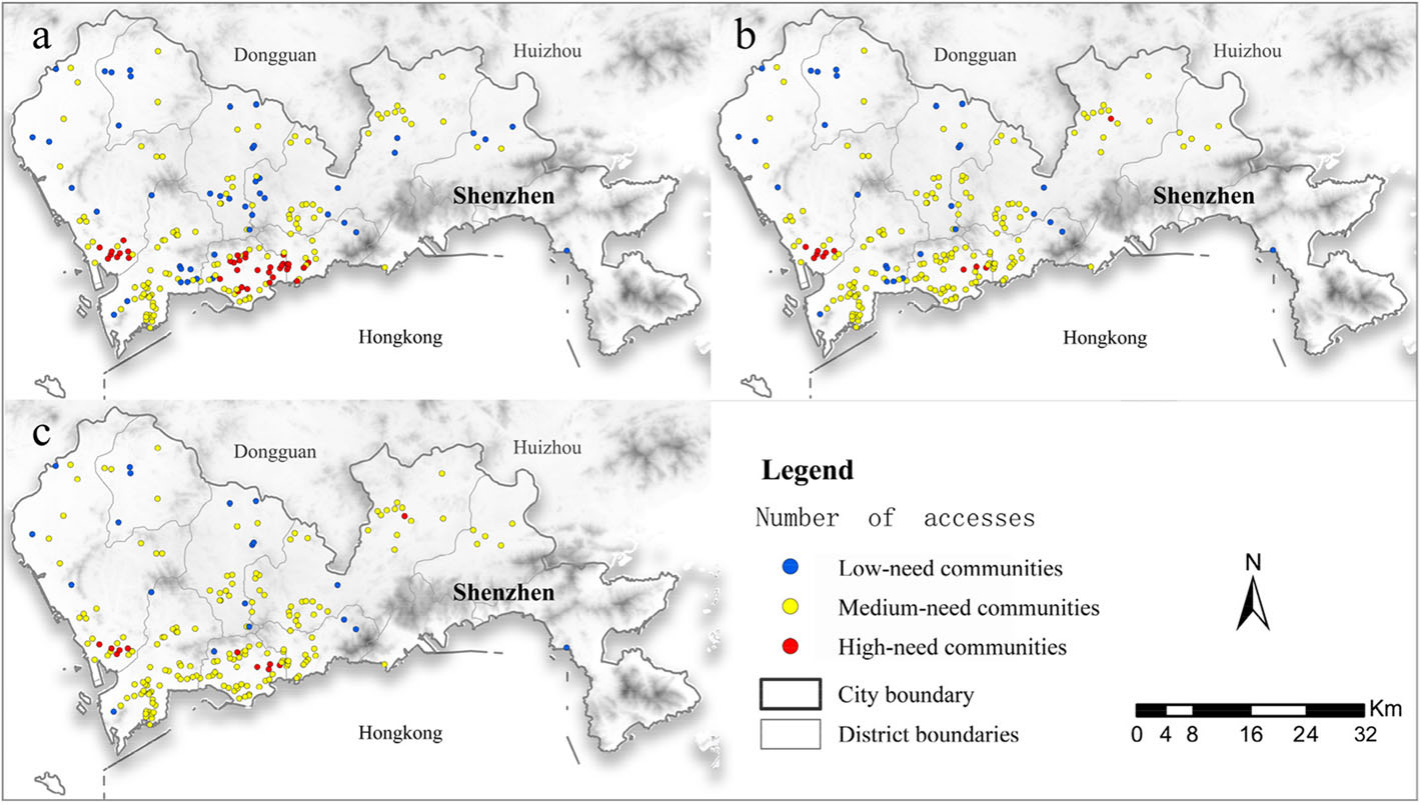
**a.** The current scheme: based on community groups with 49 FCs, there are 122 medium-need communities, 45 low-need communities, and 37 high-need communities. **b.** Scheme 1: based on community groups with 32 secondary and above hospitals as the FCs, there are 165 medium-need communities, 28 low-need communities, and 11 high-need communities; **c.** Scheme 2: based on community groups with 35 primary and above hospitals as the FCs, there are 174 medium-need communities, 19 low-need communities, and 11 high-need communities

Next, based on the cumulative opportunities of visiting FCs within the ECs in 30 min, 3 social groups can be captured (Fig. 5). Among them, the community with 0 opportunities of visiting in 30 min is a “low-need community” (22.06%), scattered in every district, the average travel time for them is 42 min. In communities with no greater than 3 cumulative opportunities of visiting FCs, “medium-need communities” account for 59.8% with 20 min of average travel time. High-need communities are concentrated in Futian, Baoshan, and Luohu District, accounting for 18.14%, with 4 or more cumulative opportunities of visiting FCs and the 15 min of average travel time. This illustrates that 40.2% of FCs need to be further optimized to improve medical accessibility and reduce transmission of COVID-19.

### Optimized allocation of FCs and effect evaluation

Considering that there are more than 218 hospitals and 661 community health centers in Shenzhen that have not been included as FCs for the coronavirus epidemic, combined with that current FCs need to return to normalize its medical services as the outbreak is stable, it is practical to dynamically adjust the distribution of the fever clinics. The LSCP (Location Set Covering Problem) model [38, 43] which attempts to locate a minimum number of servers to cover all demand nodes within the time standard, can achieve the goal of optimizing the rational and balanced use of medical resources and medical equity. Accordingly, we propose two optimization schemes: (i) adding secondary and above hospital as alternatives, (ii) adding primary and above hospital as alternatives. In order to make maximum utilization of medical resources, if the new scheme cannot completely cover the current low-need ECs during the dynamic adjustment process, we will set community hospitals to provide medical services for it. Afterward, we calculated the minimum arrival time-based on the Baidu map API, and finally build the LSCP model to obtain two allocation schemes of FCs.

Table 1 shows that compared with 49 FCs before adjustment, the number of FCs in schemes 1 and 2 have become 32 and 35, respectively. Among them: on the basis of guarantee accessibility, the number of tertiary and above hospitals has decreased by 22 and 18 respectively, which has played an active role in getting medical order to normal.

**Table 1:** Allocation schemes of FCs and comparison of changes in coverage levels

	Items	Current scheme	Scheme 1	Scheme 2
Hospitals levels with FCs	Tertiary	35	17	13
	Secondary	10	14	10
	Primary	2	1	12
	Non-level	2	0	0
	Community hospitals	0	28	19
Low-need community	number	45	28	19
	travel time	37	42	38
Medium-need community	number	122	165	174
	travel time	19	20	16
high-need community	number	37	11	11
	travel time	16	15	14

Specifically, there are 122 medium-need communities in the current scheme, with a coverage rate of only 59.8% and the average travel time is 22.42 min (Fig. 5a). After the optimization that retaining 21 original FCs and adding another 11 secondary and above hospitals in scheme 1 (Fig. 5b), the coverage rate of hospitals in medium-need and high-need communities are up to 80.88%, and the average time of going to the FCs is 22.75 min; In the scheme 2(Fig. 5c), only 15 original FCs are reserved while 20 hospitals are newly added. As a result, the coverage rate of hospitals in medium-need and high-need communities reach 85.29%, and the average time of going to the FCs is 17.94 min. To sum up, by adding secondary and above hospitals as alternatives, scheme 1 can improve the fairness of visiting in ECs, transferring the high- and low-need communities to medium-need communities; By choosing hospital at a low level such as primary and above hospitals, scheme 2 can achieve a higher coverage rate, reduce the travel time and improve accessibility.

In addition, for the ECs that can’t be covered, 28 and 19 community hospitals are required to provide services, suggesting that primary medical institutions should play a positive role in confronting the virus. Wuhan, the center of the epidemic in China, has adopted similar measures to achieve rapid screening of patients with fever or respiratory symptoms.

Apropos of the COVID-19, the allocation of medical resources must consider the balance and difference between different regions. Inequality in health care comes at the expense of individuals and society, and this will be reflected in inequality in the outcome of the epidemic [44]. Figure 5a demonstrates that high-need communities are mainly distributed in Bao’an, Futian, and Luohu Districts, which are in the core urban areas of Shenzhen. In contrast, low-need communities are usually located in borders of districts lacking medical resources. In the meantime, the migrant worker after the lunar new year in 2019 in Shenzhen is the highest, accounting for 4.54%, and it will experience large population movements. So that may result in the recurrent spread of the COVID-19 if the medical accessibility of suspected cases is not effectively improved. Through the dynamic adjustment of FCs, the gap between medical accessibility in high-need and low-need communities has been greatly reduced. Supported by the active involvement of primary medical institutions, the ECs that cannot be covered within 30 min have been effectively covered, which is necessary for the disadvantaged communities in the junction of the districts. During the rapid spread of the epidemic, many cities have made an immediate reaction and quickly provide a large number of fever clinics (for example 392 FCs in Chengdu and 426 FCs in Chongqing). In addition, these cities are dynamically optimizing and moderately reducing the FCs according to the situation of the epidemic (for example, the number of FCs in Beijing has been adjusted from 89 to 76).

## Discussion

Through this study, we show that if using the non-traveling mode, there are more than 75% of residents in Shenzhen have lower access to treatment. According to the statistical results, 45 Low-need communities (22.06%) were unable to reach the nearest fever clinic during the 30-min in non-driving mode, mainly in Nanshan District (9), Longhua District (9), and Longgang District (9). In contrast, about 60% of the FCs in Shenzhen are located in Futian, Longgang, and Baoan districts. On the whole, there is an unbalanced distribution of ECs and fever clinic resources in various districts. For example, Nanshan District has the largest number of confirmed cases while the average percentage of FCs in the epidemic community is the lowest, only 0.1, followed by Longhua District (0.17) and Luohu District (0.19); In addition, the data show that 91.8% of FCsof secondary and above hospitals, mainly concentrated in Futian District, Longgang District and Baoan District. As a result, these facilities can not achieve equitable access to medical treatment for each district and county, and even more difficult to address the issue of medical coverage for patients at the community level.

In order to overcome the above problems about unbalanced resource allocation, the LSCP model was built to realize the dynamics of FCs in Shenzhen, and two effective solutions were proposed based on the selection of different levels of alternative FCs. To be more exact, choosing secondary and above hospitals as alternatives in scheme 1 can reduce the difference between different community groups and effectively increase the coverage of ECs to 80.88%, but it can't effectively reduce the average time available for residents to visit FCs. However, selecting primary and above hospitals as alternatives can decrease the average travel time by 17.94 minutes while reducing the differences in community groups, improving medical accessibility. In addition, the proportion of tertiary hospitals in scheme 2 was reduced from 71.43% to 24.07%, reducing the overutilization of high-quality medical resources. Therefore, in the context of the current epidemic and other future public health crises, a community-wide, well-resourced, health-equitable primary health care system is not only a central policy of the national health system and emergency response [45] [29], but also a basic safety guarantee for the sustainable development of people’s lives and health.

With the widespread of an epidemic all over the world, there is an increasingly urgent health care response to the epidemic in various countries, particularly in developing countries with low efficiency of health systems [45, 46]. For example, in densely populated countries with inadequate medical resources, such as Brazil, India, and Bangladesh, the epidemic has spread to low-income groups in villages, shanty towns and favelas, and slums who work in dense, unplanned areas and cannot be provided with basic public facilities and services, including public health systems [47–49]. Thus, grass-root people are fighting with chaos, hunger, and death during the outbreak. We conclude that, given the inadequate and unbalanced allocation of medical resources in most countries as well as lacking scientific and effective guidance for the allocation of FCs, if the FCs are allocated based on experience alone, it may cause the second wave.

Therefore, based on the optimal allocation of FCs, several measures are proposed for developing and less developed countries with severe epidemic: (1) Firstly, according to the susceptible population (e.g. the elderly, low-income groups) or where the susceptible population is gathering, the current hospitals (including public hospitals and private hospitals) are selected as alternative hospitals based on the principle of visiting the nearest FCs. Then, based on the principle of maximum coverage, the FCs are distributed for rapid screening and classification of patients during early-stage. (2) Temporary mobile FCs should be established as soon as possible in areas with scarce resources and high risk, reducing the difference in medical accessibility between different communities and shorting the travel time for patients. (3) In terms of setting up FCs, the basic medical resources such as community hospitals and private clinics should be utilized and transformed as much as possible, so as to reduce the excessive occupation of high-quality medical resources and restore normal medical order as soon as possible. (4) Lastly, government should dynamically adjust and balance the allocation of FCs according to the direction of the spread of the epidemic and new changes in communities and areas.

### Limitations

Still, several limitations need to be mentioned. One limitation is that Shenzhen has not yet announced the number of confirmed and suspected cases in the epidemic community which can’t allow us to track the severe situation of the epidemic and determine the supply and demand matching relationship between patients (within the hospital’s service area) and hospital beds (or medical workers). So that we can’t observe the contradiction between the supply and demand and can’t explore plans for the deployment of new resources. Another limitation is the inadequate description of the accessibility evaluation index of visiting FCs in the epidemic community. We only considered that long-term travel will increase the risk of infection, but did not fully consider factors such as transfers times, the density of transfer hubs, and the patient’s attributes (income, age, and degree of illness, etc.), which will have a certain impact on the risk of exposure to the virus and behavior of patients.

## Supplementary Information

**Additional file 1.**

## Data Availability

The datasets used and/or analyzed during the current study are publicly available from the Shenzhen Health Commission (http://wjw.sz.gov.cn/yqxx/index.html) and Baidu Map API (http://lbsyun.Baidu.com/).

## Abbreviations

COVID-19: 2019 coronavirus disease
FCs: Fever clinics
ECs: Epidemic communities
LSCP: Location Set Covering Problem
