# Supplementary material for: Reducing exposure to COVID-19 by improving access to fever clinics: an empirical research of the Shenzhen area of China
Source: BMC Health Serv Res. 2021 Sep 13;21:959. doi: 10.1186/s12913-021-06831-4 (PMC8435565; doi:10.1186/s12913-021-06831-4)
Supplement: Supplementary file 1 — Additional file 1. [file 12913_2021_6831_MOESM1_ESM.docx]

# Supplementary Information

Reducing exposure to COVID-19 by improving access to fever clinics: An empirical research of the Shenzhen area of China

**Data source:** Publicly available at the Shenzhen Municipal Health Commission(<http://wjw.sz.gov.cn/>)

**Supplementary Fig.S1.** Daily changes in confirmed cases of "COVID-9" in Shenzhen as of February 29,2020

**Supplementary Fig.S2.** The number of ECs, confirmed cases and available beds in Shenzhen as of February 7, 2020.


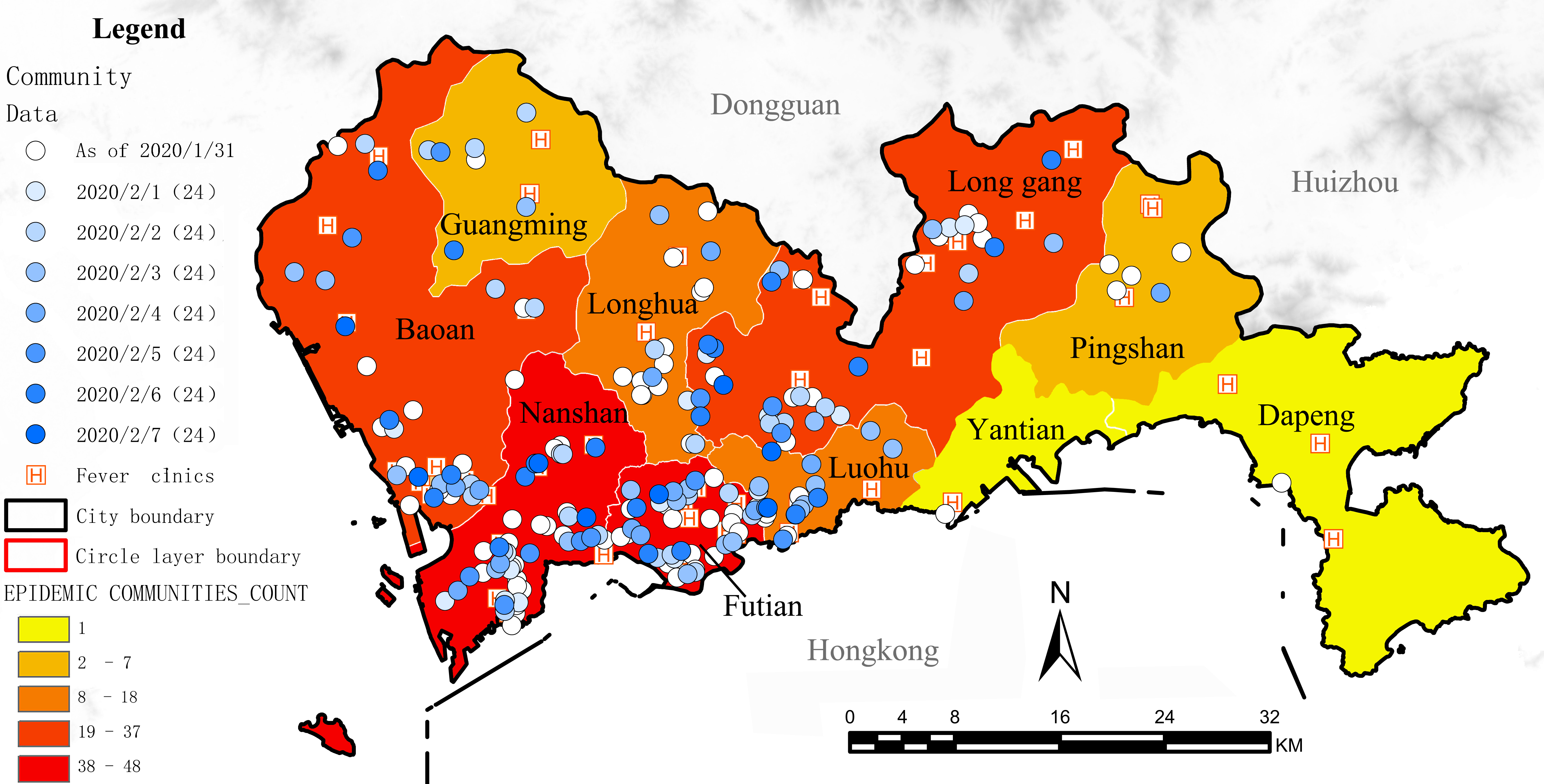


**Supplementary Fig.S3.** The spatial distribution of ECs and FCs as of February 7, 2020.

**Supplementary Fig.S4.** Baidu navigation data acquisition and process flow chart
